# Perfluorinated Compounds May Lower Vaccine Protection in Children

**DOI:** 10.1289/ehp.120-a150a

**Published:** 2012-04-01

**Authors:** Carol Potera

**Affiliations:** **Carol Potera**, based in Montana, has written for *EHP* since 1996. She also writes for *Microbe*, *Genetic Engineering News*, and the *American Journal of Nursing*.

Perfluorinated compounds (PFCs), commonly found in nonstick cookware, food packaging, and stain-repellant clothing and furniture, may lower the potency of childhood immunizations, according to a new study of residents in the Faroe Islands. Children with high blood levels of PFCs had lower antibody levels for diphtheria and tetanus than children with lower PFC levels. In some cases the antibody loads were likely too low to protect children against these infections.^1^

**What are perfluorinated compounds?**

Perfluorinated compounds, or PFCs, are water- and grease-resistant industrial chemicals used in coatings for paper plates, food packaging, rainwear, upholstery, nonstick pans, microwave popcorn bags, and lubricants for skis and snowboards.^9,10,11^ Two of the better known PFCs are perfluorooctane sulfonate (PFOS) and perfluorooctanoic acid (PFOA). People can ingest PFCs in food and water and inhale them in dust, although it is unknown which route(s) contribute the most to exposure.^12^ To limit exposure, Grandjean recommends avoiding microwave popcorn, stain repellents for furniture and carpets, and clothing and household items treated with PFCs; such items are often labeled “stain-resistant,” “nonstick,” or “stick-resistant.”

The study participants were part of a longitudinal birth cohort born in the Faroe Islands between 1997 and 2000. Researchers measured tetanus and diphtheria antibodies in 587 children at ages 5 and 7 years. The children had been vaccinated against these diseases in accordance with the standard Danish/Faroese vaccination schedule. The children’s PFC exposure was estimated from blood samples provided by their mothers soon before delivery as well as blood samples provided by the children themselves at age 5 years (children can be exposed to PFC prenatally^2^ and after birth^3^).

Both prenatal and childhood PFC exposures were associated with impaired antibody levels. At age 5, a doubling in prenatal levels of the PFC perfluorooctane sulfonate was linked to a 39% reduction in diphtheria antibodies. At age 7, a doubling in PFCs measured 2 years previously was associated with a 49% reduction in combined tetanus and diphtheria antibodies. A quarter of the 5-year-olds fell below the level of antibodies considered clinically protective for tetanus, and 37% were below the cutoff for diphtheria antibodies.^1^ Children with low antibody levels were revaccinated to boost their protection, says study leader Philippe Grandjean, an adjunct professor of environmental health at the Harvard School of Public Health.

Although the study examined only two of the many vaccines children receive, the results suggest that PFCs may dampen the anticipated lifelong protection of more of these mainstays of modern disease prevention. Only a few other agents, such as pharmaceuticals^4^ and ionizing radiation^5^ used in cancer therapy, have been shown to interfere with childhood immunizations as strongly as PFCs appeared to in this study, Grandjean says. Studies in mice have shown that exposure to PFCs suppresses the immune system,^6^ and the new results now connect these common environmental chemicals with immunotoxicity in humans. “We need to explore this in greater depth,” Grandjean says.

Studies of generally representative populations of U.S. women^7^ and children^8^ suggest these groups have roughly the same or slightly higher PFC levels, respectively, compared with their Faroese counterparts.^1^ “The negative impact of PFCs on childhood vaccinations also should be viewed as a potential threat to public health in the United States,” Grandjean says.

People tend to worry about gross adverse health effects of chemical exposures, such as cancer, points out Paige Lawrence, an associate professor of environmental medicine, microbiology, and immunology, and director of the Toxicology Training Program at the University of Rochester School of Medicine & Dentistry. “However, we overlook more subtle adverse effects, such as how chemicals affect our ability to fight infections or modify how well vaccines work,” she says.

Lawrence calls Grandjean’s study “groundbreaking” because it highlights how little we know about how environmental chemicals such as PFCs perturb the immune system. “The immunotoxicity of these chemicals needs a lot more attention,” she says.
